# A Call for Vigilance: Trigeminocardiac Reflex Presenting As Ventricular Bigeminy During Marginal Mandibulectomy

**DOI:** 10.7759/cureus.107789

**Published:** 2026-04-27

**Authors:** Bheemas Atlapure, Dharitri Dutta, Habib Md R Karim, Satheesh Gunashekar, Anirban Bhattacharjee

**Affiliations:** 1 Anaesthesiology, Critical Care and Pain Medicine, All India Institute of Medical Sciences, Guwahati, IND; 2 Anaesthesiology, Critical Care, and Pain Medicine, All India Institute of Medical Sciences, Guwahati, IND; 3 Anaesthesiology, Critical Care and Pain Medicine, All India Institute of Medical Sciences, Guwahati, Assam, IND

**Keywords:** cardiac arrhythmias, mandibulectomy, oral surgical procedures, trigeminal nerve, trigeminocardiac reflex, ventricular bigeminy

## Abstract

The trigeminocardiac reflex (TCR) is an autonomic reflex elicited by stimulation of the trigeminal nerve and is most commonly characterized by bradycardia and hypotension. In oral and maxillofacial surgery, peripheral TCR may occur during manipulation of the maxillary or mandibular divisions, occasionally presenting with atypical cardiovascular responses. We report a rare case of peripheral TCR manifesting as sudden severe hypertension with ventricular bigeminy during wide local excision and marginal mandibulectomy in a 69-year-old male with carcinoma of the lower alveolus. The hemodynamic disturbance coincided with the traction and suturing of the mandibular alveolar margin and was refractory to pharmacological management. Prompt release of surgical traction resulted in immediate resolution of both hypertension and arrhythmia, with no postoperative cardiac sequelae. This case underscores the importance of recognizing atypical presentations of TCR in maxillofacial procedures and highlights the need for close anesthetic-surgical communication and gentle tissue handling to prevent potentially life-threatening complications.

## Introduction

The trigeminocardiac reflex (TCR) is a well-recognized brainstem reflex characterized by sudden cardiovascular and respiratory changes, including bradycardia, hypotension, apnea, and, in severe cases, cardiac arrest. Less commonly, paradoxical manifestations such as tachycardia, hypertension, and increased gastric activity have also been reported [1]. The overall incidence of TCR is 20% of all cases, with the highest percentage occurring during mid-face trauma repair, followed by upper face reconstructive surgeries [2]. The neuro-anatomic arc of TCR is illustrated by the sensory nerve endings of the trigeminal nerve sending neuronal signals via the gasserian ganglion to the sensory nucleus of the trigeminal nerve, forming an afferent pathway of the reflex arc. This afferent pathway then continues along the short internuncial nerve fibres in the reticular formation to connect with the efferent pathway in the motor nuclei of the vagus nerve [3]. In most situations, careful monitoring and early recognition of warning signs are sufficient to interrupt the reflex and prevent serious consequences. Although rare, intraoperative ventricular arrhythmias accompanied by hypertension may represent an atypical manifestation of TCR, particularly its peripheral variant or the diving reflex. This is most often encountered during oral and maxillofacial surgeries, where manipulation of the peripheral branches of the trigeminal nerve is common [4]. We describe a case of TCR presenting as sudden hypertension with ventricular bigeminy during wide local excision and marginal mandibulectomy in an elderly patient.

## Case presentation

A 69-year-old man with carcinoma of the left lower alveolus was scheduled for wide local excision and marginal mandibulectomy. He had a past history of tonsillar carcinoma treated one year earlier with radiotherapy and six cycles of carboplatin-based chemotherapy. He was a known case of bronchial asthma and was using a salbutamol metered-dose inhaler as required.

**Figure 1:**
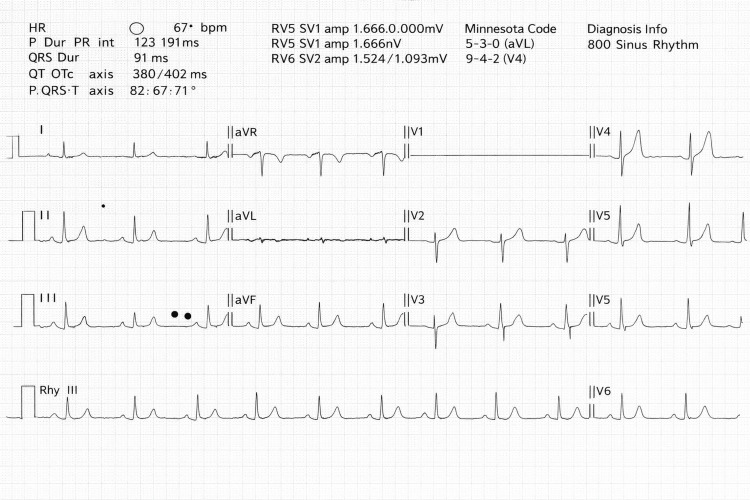
Baseline 12 lead ECG illustrating Normal Sinus Rhythm

Preoperative investigations, including baseline electrocardiography and echocardiography, were within normal limits as illustrated in Table 1 and Figure 1. He was classified as American Society of Anesthesiologists (ASA) physical status II, with a Mallampati score of II.

**Table 1:** Baseline Laboratory Investigations with Normal Reference Ranges

Parameter	Result	Unit	Reference Range
Haemoglobin (Hb)	12	g/dL	11–14
Hematocrit (Packed Cell Volume, PCV)	36	%	34–40
White Blood Cell Count (WBC)	4.96	×10⁹/L	4–11
Platelet Count	167	×10⁹/L	200–490
Random Blood Sugar (RBS)	86	mg/dL	74–106
Renal Function Tests			
Serum Sodium (Na⁺)	138	mEq/L	135–150
Serum Potassium (K⁺)	3.9	mEq/L	3.5–5.0
Serum Chloride (Cl⁻)	103	mEq/L	95–105
Blood Urea	26	mg/dL	10–43
Serum Creatinine	1.1	mg/dL	0.6–1.5
Coagulation Profile			
Prothrombin Time (PT)	12.1	seconds	10–14
International Normalized Ratio (INR)	1.02	—	1.0–1.2
Activated Partial Thromboplastin Time (aPTT)	24	seconds	20–40
Liver Function Tests			
Total Protein	6.0	g/dL	5.2–8.2
Albumin	3.7	g/dL	2.4–4.0
Globulin	2.3	g/dL	2.0–3.5
Total Bilirubin	0.4	mg/dL	0.2–1.0
Direct Bilirubin	0.3	mg/dL	0.1–0.4
Indirect Bilirubin	0.1	mg/dL	0.2–0.8
Albumin/Globulin Ratio (A/G Ratio)	1.6	—	1.1–2.5
Alanine Aminotransferase (ALT)	6	U/L	7–56
Aspartate Aminotransferase (AST)	27	U/L	8–40
Alkaline Phosphatase (ALP)	63	U/L	44–147
Gamma-Glutamyl Transferase (GGT)	22	U/L	9–48

Premedication included intravenous fentanyl 100 µg. General anesthesia (GA) was induced with propofol 2 mg/kg and vecuronium 0.1 mg/kg, with sevoflurane at 1-2% to achieve an age-adjusted minimum alveolar concentration (MAC age) of 0.5-0.7. Endotracheal intubation was performed after adequate neuromuscular relaxation. Post-induction vitals were stable, with blood pressure (BP) 120/70 mmHg and heart rate (HR) 64-70 beats/min. Mechanical ventilation was adjusted to maintain an end-tidal carbon dioxide (ETCO₂) between 30 and 38 mmHg.

General anesthesia was maintained with an oxygen-nitrous oxide mixture and isoflurane with a target MAC age of 1-1.1, with intermittent boluses of vecuronium (1 mg). Standard ASA monitoring, including ECG, non-invasive BP (NIBP), pulse oximetry, temperature, and capnography, was continued throughout the procedure.

**Figure 2:**
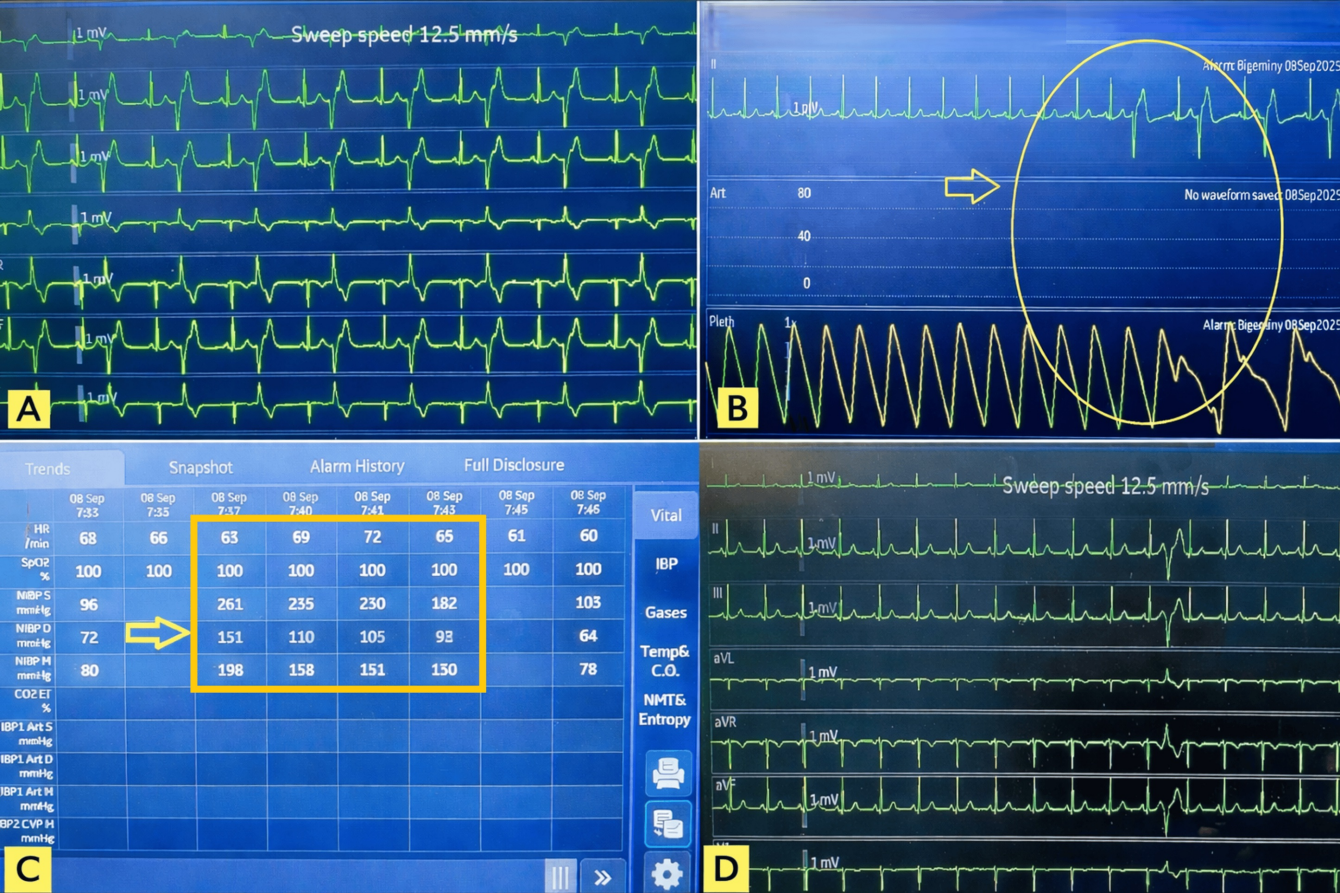
Figure illustrates the hemodynamic changes in detail upon surgical stimulation Figure 2: (A) Electrocardiogram (ECG) showing ventricular bigeminy. (B) Onset of ventricular bigeminy; the yellow arrow indicates corresponding changes in the ECG and plethysmography waveform. (C) Trend snapshot demonstrating abrupt hemodynamic changes, with the yellow arrow highlighting the boxed region showing variations in heart rate (HR) and non-invasive blood pressure (NIBP) during the episode. (D) ECG tracing showing resolution of ventricular bigeminy with return to normal sinus rhythm.

Approximately 1 hour and 20 minutes into the surgery, the patient developed sudden severe hypertension, with BP rising to 210/120 mmHg and above, followed shortly by ventricular bigeminy on ECG tracing (Figure 2A, 2B, 2C). The ECG showed ventricular bigeminy, and as the NIBP cycled every 3 minutes, the exact time frame could not be documented. Immediately on recognition, intravenous lignocaine 60 mg was added as an anti-arrhythmic to control ventricular ectopics, and propofol 20 mg was administered to augment anesthetic depth, for which there was no change in the NIBP and ECG tracing. On observation, at that time, the surgeon was suturing the exposed lower alveolar margin with moderate traction on the lower jaw. Following this, the surgical team was immediately requested to release the traction. Subsequently, on release of the traction by the surgeons, the NIBP decreased to 150/70 mmHg, and the ventricular bigeminy resolved, with restoration of normal sinus rhythm (Figure 2D). The remainder of the surgery proceeded uneventfully. The patient was extubated smoothly at the end of the procedure. Postoperative ECG did not reveal any cardiac abnormality, and the patient remained hemodynamically stable until discharge.

## Discussion

Trigeminocardiac reflex was first described in 1908 as the oculocardiac reflex byAschner and Dagnini, and later expanded to include non-ocular trigeminal stimulation by Shelley and Church, who proposed the term “TCR” [5,6,7]. TCR is an autonomic reflex that can result in significant cardiovascular disturbances during surgical procedures involving trigeminal nerve stimulation.

Based on the site of stimulation, TCR is classified into central, peripheral, and gasserian ganglionic subtypes [1]. Peripheral TCR occurs due to stimulation of the ophthalmic, maxillary, or mandibular branches of the trigeminal nerve. When the maxillary or mandibular divisions are involved, the response is sometimes referred to as the maxillomandibular cardiac reflex, which is considered a subset of TCR. The diving reflex, one of the most powerful autonomic reflexes, is regarded as a modified peripheral form of TCR [8].

Central TCR is typically triggered by stimulation of the intracranial portion of the trigeminal nerve between the gasserian ganglion and brainstem nuclei and is most commonly observed during neurosurgical procedures near the brainstem [1,9]. Ganglionic TCR, arising from direct stimulation of the gasserian ganglion, is associated with distinct hemodynamic responses [1,8].

Trigeminocardiac reflex has been reported during a wide range of craniofacial procedures, including skull base, ophthalmic, maxillofacial, and dental surgeries [10]. It may even occur during seemingly minor interventions such as suturing or instrument placement near a trigeminal nerve branch [10].

In the present case, sudden hypertension with ventricular bigeminy occurred during traction and suturing over the lower alveolar region, an area closely related to the mandibular branch of the trigeminal nerve. While intense vagal stimulation typically produces bradycardia and hypotension, milder or mixed autonomic stimulation can result in paradoxical hypertension [9]. Though the administration of injection lignocaine and propofol had no effect, the prompt resolution of arrhythmia and hypertension following release of traction supports the diagnosis of peripheral TCR. However, one can argue that the significant increase in blood pressure can be due to pain or administration of sympathomimetics, but in our case, the patient was preemptively administered injection paracetamol 1 g IV infusion and also O_2_:N_2_O as an inhalation mixture, and no sympathomimetic drugs were administered by the anesthesiologist, nor any local instillation of adrenaline at the surgical site was done by the surgeon. The depth of anesthesia was maintained with an age-adjusted MAC (MAC age) of isoflurane between 0.5 and 0.7 throughout. Earlier, the use of halothane triggered premature ventricular complexes by sensitizing the myocardium to the circulating catecholamines, which is not so in our case. Also, hypokalemia can sometimes trigger arrhythmias, causing ventricular bigeminy-associated ST segment depression, T wave flattening/depression, and prominent U waves, which were not present in our case [11].

Hypoxia and hypercapnia can also trigger arrhythmias, but it was unlikely in our case, as pulse oximetry and capnography monitoring were maintained within normal limits. One argument can be that such severe hypertension can trigger myocardial demand mismatch, as elevated blood pressure compels the heart to exert more effort to circulate blood against heightened resistance. If long-standing, it can lead to raised intramural pressure leading to subendocardial ischaemia, triggering ventricular bigeminy, which can sometimes be reflected as T-wave inversion in ECG tracing, which was not present in our case [12].

The sub-endocardium, the heart muscle's innermost layer, is particularly susceptible to oxygen deficiency. This vulnerability arises because it is the last to receive blood flow and experiences the highest pressure during systole. When the heart's oxygen demand exceeds supply, this layer can be deprived of oxygen. Such ischemia can irritate the heart muscle cells, potentially causing arrhythmias. On an electrocardiogram (ECG), T-wave inversions often indicate myocardial ischemia or damage [12,13]. However, ischemia can also occur with normal ECG readings or present with other changes, such as ST-segment depression, which was not observed in our case.

Although TCR classically presents with hypotension, hypertension may occur due to peripheral vasoconstriction resembling a diving reflex response, heightened sympathetic activity from surgical pain, or relatively inadequate anesthetic depth. Known risk factors for TCR include hypoxia, hypercapnia, light anesthesia, younger age, opioid use, beta-blockers, calcium channel blockers, acidosis, and the intensity and duration of the stimulus [4]. In this case, anesthetic depth and ventilation were adequate, suggesting that mechanical traction at the surgical site was the primary trigger. Careful surgical technique with minimal traction, heightened vigilance during procedures near trigeminal branches, and, where feasible, the use of nerve monitoring may help reduce the risk of TCR [3,4,14].

Prophylactic strategies for the TCR should prioritize modified surgical techniques, specifically gentle, gradual manipulation as the primary approach, with pharmacological measures such as anticholinergics or local anesthetics acting as secondary, adjunctive measures. The first-line response to a suspected TCR is the immediate cessation of surgical manipulation. Preemptive local anesthetic infiltration (e.g., lidocaine) in the area of sensory nerve distribution before manipulation is considered a highly effective proactive modified technique for blocking the afferent pathway. Although intravenous atropine or glycopyrrolate can be used to treat TCR-induced bradycardia, their prophylactic use is controversial [15]. High doses of prophylactic atropine have not been sufficient to completely prevent TCR in some studies and can cause adverse arrhythmias. It is most useful to have anticholinergics prepared for immediate administration if the pulse rate drops rapidly rather than relying solely on preemptive administration [16]. However, due to the absence of bradycardia in this case, the administration of atropine or glycopyrrolate would have been detrimental to the patient by significantly increasing the heart rate and blood pressure.

## Conclusions

This case highlights a rare and atypical presentation of trigemino-cardiac reflex as ventricular bigeminy with hypertension during oral surgery. TCR can contribute to significant perioperative hemodynamic instability and potential complications of cardiac arrest and asystole. A combination approach is optimal, but the surgical technique is superior. Surgeons should be aware of high-risk moments and utilize gentle, gradual manipulation. Vigilant communication between the surgical and anesthetic teams is paramount to anticipate the reflex. Early recognition, prompt removal of the triggering stimulus, and appropriate anesthetic management are crucial. Awareness of such uncommon presentations and continuous ECG monitoring are essential to ensure patient safety during procedures involving trigeminal nerve manipulation.
